# Severity of irritable bowel syndrome in patients with temporomandibular disorders: A case-control study

**DOI:** 10.4317/jced.55649

**Published:** 2019-09-01

**Authors:** Nicola Mobilio, Paola Iovino, Vincenzo Bruno, Santo Catapano

**Affiliations:** 1 DDS, Research Assistant, Dental School, Dental Clinic, University of Ferrara, c.so Giovecca, 203, 44121 Ferrara, Italy; 2 MD, Associate Professor, Functional GI Disorders Center at Gastrointestinal Unit, AOU S. Giovanni di Dio e Ruggi d’Aragona, Department of Medicine and Surgery, University of Salerno, v. S. Allende, Baronissi, 84084 Salerno, Italy; 3 MD, DDS, Lecturer, Dental School, Dental Clinic, University of Ferrara, c.so Giovecca, 203, 44121 Ferrara, Italy; 4 MD, DDS, Associate Professor, Dental School, Dental Clinic, University of Ferrara, c.so Giovecca, 203, 44121 Ferrara, Italy

## Abstract

**Background:**

To assess the risk and severity of IBS in a population of TMD patients.

**Material and Methods:**

Subjects for the study group were recruited from patients attending the Dental Clinic. Health controls (HC) were recruited among patients’ friends and clinic staff. All subjects filled in the RDC/TMD questionnaire and the ROME III questionnaire for the diagnosis of IBS. The IBS Severity Scoring System (IBS-SSS) was used to evaluate the severity of each case of IBS. Categorical variables were compared through the Chi square test. The risk of having abdominal pain was analysed using logistic regression.

**Results:**

Twenty-two (46.8%) cases of IBS were diagnosed among TMD patients, whereas only 4 (11.4%) were in the HC group. This difference was statistically significant (χ2(1)=11.6; *p*<.01). The differences in the distribution of IBS-SSS were statistically significant (χ2(3)=12.49; *p*<.05). The regression model resulted statistically significant (χ2(5)=24.08; *p*<.001, R2=.37): abdominal pain was significantly related to nonspecific physical symptoms independent of the other variables.

**Conclusions:**

TMD patients had a greater risk of having IBS compared to HC. TMD patients presented also more severe form of IBS than HC.

** Key words:**Temporomandibular disorders, irritable bowel syndrome, facial pain, case-control study.

## Introduction

Temporomandibular disorders (TMD) include different clinical conditions that involve masticatory muscles and/or temporomandibular joints (TMJs) (1) presenting common symptoms: pain in the area of jaw muscles and/or the TMJ; limitation or alteration of mandibular movements and TMJ sounds (2). TMD patients often complain symptoms in other body regions and meet diagnostic criteria for other clinical conditions. Associations were found between TMD and migraine and chronic fatigue syndrome (3), fibromyalgia (4), vulvar vestibulitis syndrome (5), anxiety and depressive disorders (6), irritable bowel syndrome (IBS) (7–9) and others. Many of these disorders are considered as central sensitivity syndromes (CSS), in which the phenomenon of central sensitization leads to an increase of the excitability of neurons in central nociceptive pathways and an inhibition of the descending pain modulatory system (10). Muscular subtypes of TMD (i.e. group I of RDC system (11) show typical aspects of CSS (3).

IBS is a chronic functional disorder of the lower gastrointestinal tract characterized by a group of symptoms including chronic abdominal pain or discomfort associated with altered bowel habits (12). Prevalence of IBS was estimated on 11% of the global population. Women are two to three times more likely to be diagnosed with IBS than men (13). People diagnosed with IBS are usually younger than 45 years old. IBS was found associated to other clinical conditions, like other gastrointestinal functional syndromes, migraine, chronic fatigue syndrome, chronic pelvic pain, fibromyalgia, depressive syndromes and anxiety disorders (14–17). Recently, a strong correlation was found between TMD and IBS (18): a sample of IBS patients showed a more than three times greater risk of TMD compared to controls. In that study, no differences in IBS severity was found between patients with or without TMD diagnosis. Furthermore, no previous studies investigated the severity of IBS in TMD patients. The aim of the present study was twofold: 1) to assess the risk of IBS in a population of TMD patients and 2) to assess the severity of IBS in the same population.

## Material and Methods

Subjects for the study group were recruited from patients attending the Dental Clinic of the University of Ferrara. The inclusion criterion was the presence of at least one TMD diagnosis according to RDC criteria (11). Patients with other orofacial pain diagnosis were excluded. Health controls (HC, i.e. without signs and symptoms of TMD) were recruited among patients’ friends and clinic staff. Enrolment took place between June 2016 and January 2017. All subjects filled in the RDC/TMD questionnaire (11) and the ROME III questionnaire for the diagnosis of IBS (12). According to RDC/TMD questionnaire, TMD diagnosis was obtained for both the physical and psychosocial axis, Axis I and Axis II, respectively. For Axis II, the severity of chronic pain was measured using the Graded Chronic Pain Scale (GCPS), allowing the categorization of five levels of pain–related impairment. Depression and somatization levels were evaluated by depression and somatization scales from the 90R Symptom Checklist (SCL-90R). According to that, patients were classified as having normal, moderate or severe impairment levels according to depressive and non-specific physical symptoms. Every possible diagnosis of IBS was identified through the filling of a specific questionnaire and considering the ROME III criteria. The IBS Severity Scoring System (IBS-SSS) was used to evaluate the severity of each case of IBS. This widely accepted scoring system considers the following variables: current abdominal pain intensity measured through a 0-100 visual analogue scale (VAS) and the frequency of abdominal pain; current abdominal distension expressed through VAS; intestinal habits satisfaction through VAS; degree of interference by IBS pathology in work and normal social activities through VAS. Summing single VAS scores allowed to classify IBS patients into three groups of severity: mild (75 to 175), moderate (175 to 300) and severe (> 300).

The study protocol was approved by the local Ethics Committee, it is in compliance with the Helsinki Declaration and all subjects gave written informed consent.

A sample of 79 was calculated for a confidence level of 95% and confidence interval of 10.

Categorical variables were compared through the Chi square test. The post-hoc comparison (when applicable) was made using z-test for multiple comparisons. The risk of having abdominal pain was analysed using logistic regression. *P* <.05 was considered as statistically significant. SPSS for MAC OS X version 24.0 (SPSS, Chicago, IL, United States) was used for data processing and analysis.

## Results

Forty-seven TMD patients (42 females, mean age 49.94+/-13.76 years) and 35 healthy controls (HC) (29 females, mean age 42.40+/-11.44 years) were enrolled in the study.

Following the ROME III criteria for the diagnosis of IBS, 22 (46.8%) cases of IBS were diagnosed among TMD patients, whereas only 4 (11.4%) were in the HC group. This difference was statistically significant (Chi square (1)=11.6; *p*<.01). TMD group had a greater than six times risk of having IBS compared to HC (OR=6.82, 95% CI:2.08-22.38).

The difference in the distribution of GCPS between TMD and HC groups was statistically significant (Chi square (4)=53.62, *p*<.001). There were significant differences in the distribution of GCPS grade 0, grade II (31.9% vs 5.7%) and grade III (38.3% vs 0%). There was no difference regarding to GCPS grade I (19.1% vs. 5.7%) and Grade IV (2.1% vs. 0.0%).

No statistically significant difference emerged in the distribution of depression scores between TMD and HC. The difference in the distribution of nonspecific physical symptoms (pain items included) between TMD and HC groups was statistically significant (Chi square (2)=11.14; *p*<.05). There were significant differences in the distribution of normal (17.0% vs 37.1%) and severe levels (59.6% vs. 22.9%). There was no difference for moderate levels (23.4% vs. 40.0%). The difference in the distribution of nonspecific physical symptoms (pain items excluded) between TMD and HC groups was statistically significant (Chi square (2)=11.83; *p*<.05). There were significant differences in the distribution of normal (21.3% vs 45.7%) and severe levels (57.4% vs. 20.0%). There was no difference for moderate levels (21.3% vs. 34.3%).

**Figure 1:**
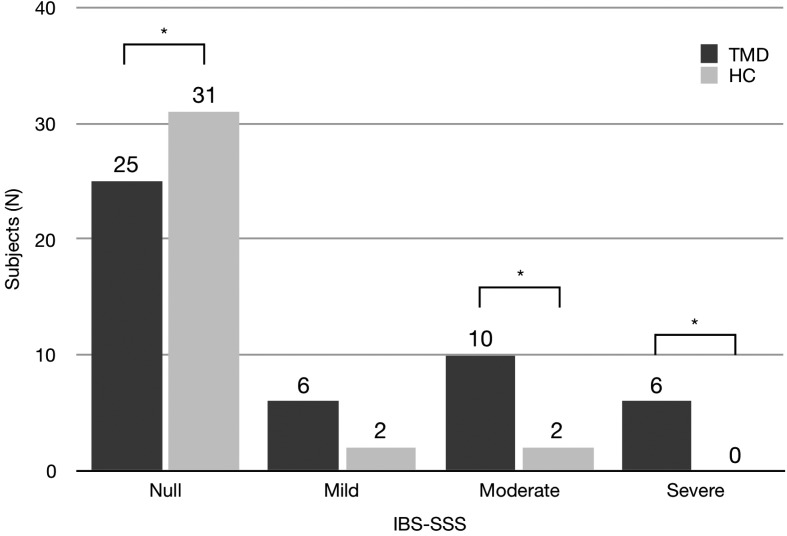
Distribution of subjects according to GCPS score. Asterisks indicate significance differences (Chi square, *p*<.05).

The severity of IBS, according to IBS-SSS score, is reported in Fig. 1. The differences in the distribution of IBS-SSS were statistically significant (Chi square (3)=12.49; *p*<.05). There were significant differences in the distribution of moderate (21.3% vs 5.7%) and severe IBS-SSS (12.8% vs 0%). On the other hand, there was no difference for mild IBS-SSS (12.8% vs. 5.7%).

**Figure 2:**
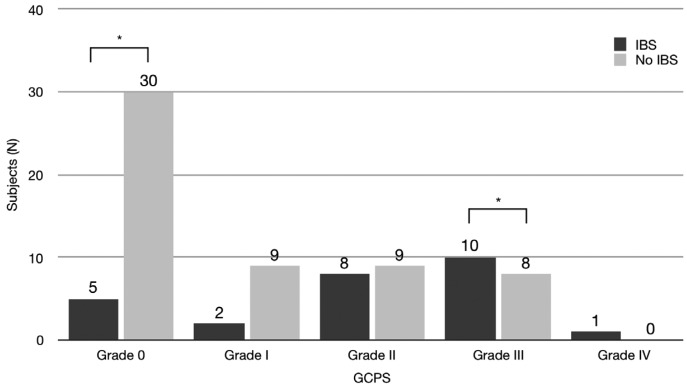
Distribution of subjects according to depression score.

**Figure 3:**
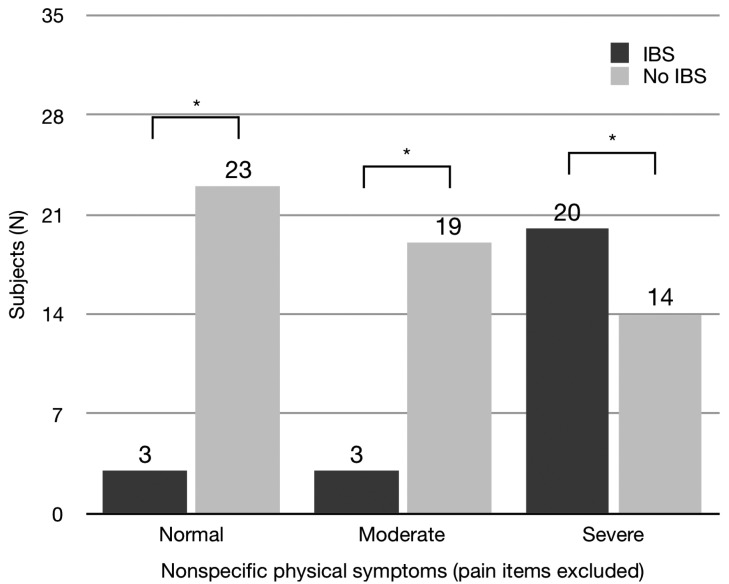
Distribution of subjects according to nonspecific physical symptoms (pain items included). Asterisks indicate significance differences (Chi square, *p*<.05).

The difference in the distribution of GCPS between subjects with and without IBS was statistically significant (Chi square (4)=14.57, *p*<.01, Fig. 2). There were significant differences in the distribution of GCPS grade 0 and grade III. No difference was found for depression. The difference in the distribution of nonspecific physical symptoms (pain items included) between subjects with and without IBS was statistically significant (Chi square (2)=13.74; *p*<.01). There were significant differences in the distribution of normal and severe levels. The difference in the distribution of nonspecific physical symptoms (pain items excluded) between subjects with and without IBS was statistically significant (Chi square (2)=19.75; *p*<.001, Fig. 3). There were significant differences in the distribution of all levels.

There was a significant correlation between the presence of facial pain and the presence of abdominal pain. However, when regression model was performed, abdominal pain was significantly related only to physical symptoms (Chi square (5)=24.08; *p*<.001, r square 2=.37,  Table 1).

**Table 1:**
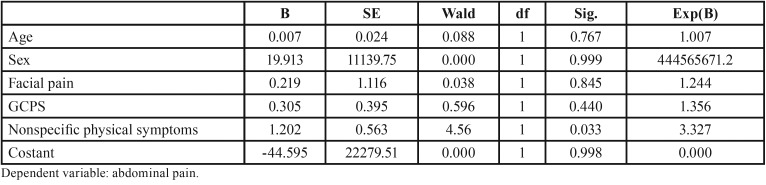
Logistic regression analysis: abdominal pain by age, sex, facial pain, GCPS and nonspecific physical symptoms.

## Discussion

The present results showed that TMD patients had a risk of suffering of IBS more than six times greater than HC. Analysis for subgroups of TMD diagnosis was not performed because of the small number of subjects in some subgroups (the majority of TMD patients being muscular). For Axis II, as expected, TMD patients showed higher levels than HC for all the variables (with the exception of depression).

A difference was found for the distribution of IBS severity in TMD patients. Both moderate and severe forms of IBS were more frequent in TMD patients rather than HC. That means that TMD patients not only have a higher risk to suffer from IBS, but that IBS is present in more severe form in TMD patients. While the higher risk of IBS in TMD patients has been established previously (3,9), this is the first time that such a correlation was found between TMD and IBS severity. These results underlined the complexity of a complete diagnosis in TMD patients: they often present symptoms related to body regions different from orofacial one (19). This consideration lead to clinically important consequence: a TMD patient can suffer from other diseases that can be overlooked, not diagnosed and then not treated (8,20).

The analysis of the distribution of Axis II variables according to the diagnosis of IBS revealed some interesting aspects. Excluding depression (that was not significant), both GCPS and physical symptoms showed significant differences. Subjects with IBS showed higher level of chronic pain and physical symptoms than subjects without IBS. Such results underlined the psychosocial involvement of IBS subjects, and are similar and “symmetrical” to previous results (18).

Regression analysis for abdominal pain also revealed something interesting: after including Axis II variables, the only predictor of abdominal pain was nonspecific physical symptoms. Such a result suggests that rather than TMD patients being more prone to IBS (or vice versa), it is possible that such patients suffer from a general, systemic disorder that expresses in different clinical forms. Such a systemic disorder may involve different body regions and systems: in the orofacial region we call it “TMD”, in intestinal region we call it “IBS”. It is not the first time that the hypothesis of CSS is proposed for TMD (10). These results underline one more time the importance of routinely using Axis II questionnaires: just clinical diagnosis is not enough for completely understanding a TMD patient, but psychosocial evaluation is mandatory. Investigating the overlapping of different symptoms and disorders may dramatically help the management of chronic pain patients: indeed, a simultaneous approach has been proved to be more effective than single therapies (21). Further studies should deeply investigate these not yet completely cleared aspects.

This study presents some limits. Even if the two samples are homogeneous for age and gender distribution, they both have a small number of subjects. The present design is a case-control study that is suitable to find an association but not a cause-effect relationship. Further studies with more enrolled subjects and a different design (i.e. cohort study) are needed to overcome these limits.

However, within the above mentioned limits, it is possible to conclude that:

TMD patients present a greater risk of having IBS compared to controls;

IBS presents in more severe forms in TMD patients rather than controls;

For a complete evaluation of TMD patients, it is crucial to perform Axis II diagnosis.
